# Cardiovascular Mortality Risk After Cancer Diagnosis by County‐Level Characteristics in the United States 2000–2021

**DOI:** 10.1002/cam4.71675

**Published:** 2026-02-25

**Authors:** Yenan Zhu, Ryan Suk, Yueh‐Yun Lin, Young‐Rock Hong, Beth Virnig

**Affiliations:** ^1^ Department of Health Services Research, Management and Policy, College of Public Health and Health Professions University of Florida Gainesville Florida USA; ^2^ UF Health Cancer Center Gainesville Florida USA; ^3^ Nell Hodgson Woodruff School of Nursing Emory University Atlanta Georgia USA; ^4^ Department of Health Policy and Management, Rollins School of Public Health Emory University Atlanta Georgia USA; ^5^ Winship Center for Cancer Health Equity Research, Winship Cancer Institute Emory University Atlanta Georgia USA; ^6^ Center for Health Data Science & Analytics, Houston Methodist Academic Institute Houston Texas USA; ^7^ Department of Family and Preventive Medicine, School of Medicine Emory University Atlanta Georgia USA; ^8^ College of Public Health and Health Professions University of Florida Gainesville Florida USA

**Keywords:** cancer survivorship, cardiovascular mortality, geographic disparities, health disparities, rural health

## Abstract

**Background:**

Advances in cancer therapy have improved survival, but cardiovascular disease (CVD) is now the leading non‐cancer cause of death among survivors. Specialized cardio‐oncology care mitigates risk, yet access remains limited outside of urban academic centers.

**Methods:**

We conducted a retrospective cohort study using Surveillance, Epidemiology, and End Results (SEER) 17 Registries (2000–2021), including 6,467,098 individuals with first primary malignancies. Outcomes were CVD‐specific mortality, estimated using standardized mortality ratios (SMRs) and excess absolute risks (EARs). Exposures were county‐level urban/rural status, persistent poverty status, and racial composition. Cancer‐specific analyses were additionally performed for major cancer sites to assess heterogeneity in county‐level disparities.

**Results:**

During follow‐up, 394,540 CVD deaths occurred (SMR = 1.11; 95% CI = 1.11, 1.11). Survivors in rural counties (SMR = 1.27), counties with persistent poverty (SMR = 1.35), and those with the highest quartile of Black residents (SMR = 1.15) had significantly higher CVD mortality compared with the general population. The highest risk was observed in rural counties with persistent poverty (SMR = 1.53). Across county groups, CVD mortality peaked within the first year after diagnosis and remained elevated for over a decade in disadvantaged communities. Substantial heterogeneity was found across cancer types in county‐level disparities in CVD mortality, with the largest EAR differences observed among survivors of lung and bronchus cancer, followed by corpus uteri, prostate, and urinary bladder cancers.

**Conclusions:**

Cancer survivors experience substantial and sustained excess CVD mortality, with the greatest disparities in rural and persistently impoverished counties. These findings highlight the need to integrate cardiovascular surveillance into survivorship care and expand access to cardio‐oncology services in socially vulnerable communities.

## 1 Introduction

The population of cancer survivors in the United States (US) is projected to grow nearly 50% from 18 million to more than 26 million by 2040 [1, 2]. As the number of survivors increases, optimizing long‐term care to mitigate late effects of cancer therapy and improve quality of life has become a critical priority. Cardiovascular disease (CVD) is the leading cause of non‐cancer mortality among cancer survivors [3, 4, 5]. According to a meta‐analysis study, cancer survivors are about 55% more likely to die from CVDs compared with people in the general population [6, 7, 8]. This elevated risk is driven in part by the cardiotoxic effects of treatments such as chemotherapy and radiation, which may manifest years after therapy [9, 10]. Although recognition of cardiovascular complications related to cancer therapies has grown, gaps remain in addressing geographic and structural challenges to effective survivorship care.

Specialized cardio‐oncology care, which integrates cardiologists and oncologists to manage treatment‐related cardiovascular risks, is essential for mitigating this burden [11]. However, clinics providing such care are predominantly located in urban areas and academic medical centers, limiting access for rural populations [12, 13, 14]. Cancer survivors in rural areas often face reduced availability of specialists, delayed follow‐up care, and poorer health outcomes [15, 16, 17]. Similarly, survivors residing in counties with persistent poverty experience compounded challenges [18, 19, 20]. The economic instability stemming from area‐level persistent poverty leads to systemic under‐investment in health and health‐adjacent infrastructure such as education, housing, employment, healthcare infrastructure, and preventive services [21]. The deteriorated infrastructure in less affluent areas further limits access to healthy foods, recreational facilities, and opportunities for engaging in healthy behaviors [22]. These structural barriers may independently exacerbate CVD risk beyond individual‐level factors.

Racial and ethnic differences in CVD outcomes among cancer survivors are well‐documented [23, 24]. As race and ethnicity are social constructs, these disparities likely reflect the effects of structural racism, socioeconomic disadvantage, and environmental exposures [25, 26]. For instance, Black individuals are disproportionately exposed to neighborhood poverty, environmental stressors, and systemic barriers to care [27]. In addition to individuals' race and ethnicity, area‐level racial composition or segregation may reflect broader systemic inequities in resource allocation and healthcare access.

Most studies of CVD mortality in cancer survivorship focus on individual clinical or demographic risk factors and pay limited attention to contextual determinants. While acknowledging that specific cancer treatments and types contribute significantly to individual cardiovascular risk, this study aimed to provide a broad landscape assessment of cardiovascular mortality risk among the entire cohort of US cancer survivors based on area‐level social determinants of health. Understanding geographic variation in CVD mortality by county‐level characteristics, including urban/rural designation, persistent poverty, and racial composition, could identify contextual drivers amenable to policy intervention. Using the population‐based data from Surveillance, Epidemiology, and End Results (SEER) Program 17 Registries, we aimed to estimate cardiovascular‐specific mortality risk among cancer survivors stratified by county‐level attributes. We hypothesized that rural counties, counties with persistent poverty, and counties with a higher proportion of Black residents have elevated CVD mortality rates after cancer diagnosis.

## 2 Methods

### 2.1 Data Source

We conducted a retrospective, population‐based study utilizing data from SEER 17 Registries, which encompasses information on people with cancer from 17 geographic registries across the US [28]. To facilitate comparisons with the general US population, mortality data were sourced from the US National Center for Health Statistics through SEER. Additional county‐level data on rurality and persistent poverty, defined by the US Census Bureau [29] and Department of Agriculture [30], were also obtained from the SEER 17 database. The dataset is deidentified and publicly accessible under a data use agreement with the US National Cancer Institute; the University of Florida Institutional Review Board determined that our study is exempt. This study followed the Strengthening the Reporting of Observational Studies in Epidemiology (STROBE) reporting guideline for cohort studies.

### 2.2 Study Population

Our cohort included individuals diagnosed with their first primary malignant tumor between January 1, 2000 and December 31, 2021. These individuals were followed from cancer diagnosis to the endpoint, defined as death from CVDs, death from other causes, the last known alive date, or December 31, 2021, whichever occurred first. Exclusions were made for diagnoses based solely on death certificates or autopsies and for individuals whose age at diagnosis was unknown or under 15 years.

### 2.3 Variables of Interest

The primary outcome of interest was CVD‐specific mortality, defined as death attributed to heart disease (e.g., ischemic heart disease, heart failure), hypertension, cerebrovascular disease, atherosclerosis, aortic aneurysm/dissection, and other diseases of arteries, arterioles, or capillaries (Table S1). The cause of death was ascertained using International Classification of Diseases (ICD) codes from death certificates. County‐level characteristics encompassed urban–rural status, defined by 2013 Rural–Urban Continuum Area codes, with non‐metropolitan counties (codes 4–9) considered rural and metropolitan counties (codes 1–3) urban. Racial composition, used as a proxy for residential segregation, was measured by the percentage of Black residents in each county. Counties were stratified into quartiles based on the national distribution: Q1 (0%–0.40%), Q2 (0.41%–1.98%), Q3 (1.99%–10.53%), and Q4 (10.54%–86.65%), representing increasing concentrations of Black residents. We did not restrict the quartile estimation to the 17 registries because we wanted the quartiles to be based on the nationwide distribution of county segregation level, which is more appropriate for identifying affluent counties. Following the US Department of Agriculture's Economic Research Service classification, we defined persistent poverty counties as those in which ≥ 20% of the population lived below the federal poverty line in each of four measurement periods (1990, 2000, 2007–2011, and 2015–2019) [31]. All other counties were classified as non‐persistent poverty counties [31]. Individual‐level demographic variables, including age at diagnosis and sex, were obtained from SEER sources. Individual race and ethnicity were divided into 5 groups: Hispanic (all races), non‐Hispanic White, non‐Hispanic Black, non‐Hispanic Asian/Pacific Islander (API), and non‐Hispanic other.

To explore potential contextual confounding, we also examined additional county‐level socioeconomic characteristics. Educational attainment was measured as the percentage of adults aged 25 years or older without a high school diploma, categorized into national quartiles. Median household income was also classified into national quartiles. The unemployment rate was defined as the percentage of the unemployed civilian labor force, also categorized into national quartiles.

### 2.4 Statistical Analysis

We calculated standardized mortality ratios (SMRs) by dividing the observed number of CVD deaths in the cancer cohort by the expected number in a demographically similar general population. The expected number of cases was estimated from stratum‐specific CVD mortality rates in the general population, adjusting for person‐years at risk in our study cohort. The 95% confidence intervals (CIs) for SMRs were derived using a Poisson regression model, with statistical significance set at a CI range not overlapping 1.0. Excess absolute risks (EARs) were computed as the additional number of CVD deaths per 10,000 person‐years among the cancer cohort compared with expectations. Stratified descriptive analyses were conducted to summarize the demographic characteristics of our study cohort. Then, we estimated comparative CVD risk, expressed as the SMR, by county‐level attributes including rurality, racial composition, and persistent poverty status. We also stratified by demographic factors within each county attribute to compare the risk of CVD death across demographic characteristics. To examine whether the CVD risk varied by time since cancer diagnosis, we estimated SMRs by latency period (time since cancer diagnosis) across county attributes. We repeated EAR estimation additionally stratified by major cancer type, defined as the most common first primary malignancies in the cohort (including breast, prostate, lung and bronchus, colon and rectum, urinary bladder, corpus uteri, and kidney and renal pelvis). For each cancer type, we quantified heterogeneity in county‐level disparities using the absolute difference in EARs between county attributes. All analyses were conducted using SEER*Stat software, version 9.0.31.0. Statistical tests were two‐sided, with a statistical significance threshold of *p* < 0.05.

## 3 Results

We identified 6,467,098 individuals with a cancer diagnosis residing in 616 counties. The study population was predominantly male (51.1%), non‐Hispanic White (71.2%), and aged 65 years and older (50.8%). Geographically, 87.6% of cancer survivors resided in urban counties, 45.5% lived in counties in Q3 of Black resident concentration, and 93.6% lived in non‐persistent poverty counties (Table 1). During 36,446,839 person‐years of follow‐up (median = 5.6), 394,540 CVD‐specific deaths were observed. The majority of these deaths occurred in urban counties (86.2%), counties in Q3 of Black residents (44.6%), and without persistent poverty (92.9%). The expected number of CVD deaths for this population was 355,490, yielding a SMR of 1.11 (95% CI = 1.11, 1.11; EAR = 10.7 per 10,000 person‐years).

**TABLE 1:** Baseline characteristics by county attribute, SEER 17, 2000–2021.

Characteristic	Rural/urban status^a^		Persistent poverty status		Percentage of Black residents quartile^b^			
	Rural	Urban	Persistent poverty	Non‐persistent poverty	1 (lowest)	2	3	4 (highest)
	No. (%)							
Total	798,381 (12.4)	5,667,395 (87.6)	415,048 (6.4)	6,050,728 (93.6)	148,216 (2.3)	760,545 (11.8)	2,939,155 (45.5)	2,617,866 (40.5)
Age group								
15–39 years	35,843 (4.5)	350,786 (6.2)	22,223 (5.4)	364,406 (6.0)	7078 (4.8)	43,390 (5.7)	178,305 (6.1)	157,857 (6.0)
40–64 years	328,348 (41.1)	2,467,314 (43.5)	180,149 (43.4)	2,615,513 (43.2)	57,143 (38.6)	318,384 (41.9)	1,258,074 (42.8)	1,162,063 (44.4)
65+ years	434,190 (54.4)	2,849,295 (50.3)	212,676 (51.2)	3,070,809 (50.8)	83,995 (56.7)	398,771 (52.4)	1,502,776 (51.1)	1,297,946 (49.6)
Sex								
Male	427,170 (53.5)	2,875,711 (50.7)	218,824 (52.7)	3,084,057 (51.0)	80,120 (54.1)	397,558 (52.3)	1,494,448 (50.9)	1,330,758 (50.8)
Female	371,211 (46.5)	2,791,684 (49.3)	196,224 (47.3)	2,966,671 (49.0)	68,096 (45.9)	362,987 (47.7)	1,444,707 (49.1)	1,287,108 (49.2)
Race and ethnicity								
Hispanic	28,629 (3.6)	681,620 (12.0)	45,904 (11.1)	664,345 (11.0)	2286 (1.5)	50,733 (6.7)	363,815 (12.4)	293,415 (11.2)
Non‐Hispanic								
Asian/Pacific Islander	13,854 (1.7)	448,096 (7.9)	6172 (1.5)	455,778 (7.5)	427 (0.3)	27,552 (3.6)	271,368 (9.2)	162,605 (6.2)
Black	62,516 (7.8)	599,749 (10.6)	86,305 (20.8)	575,960 (9.5)	261 (0.2)	6234 (0.8)	133,191 (4.5)	522,579 (20.0)
White	685,346 (85.8)	3,915,598 (69.1)	272,463 (65.7)	4,328,481 (71.5)	144,469 (97.5)	665,011 (87.4)	2,156,495 (73.4)	1,634,973 (62.5)
Other^c^	8036 (1.0)	22,332 (0.4)	4204 (1.0)	26,164 (0.4)	773 (0.5)	11,015 (1.5)	14,286 (0.5)	4294 (0.2)

Abbreviations: No., number; SEER, Surveillance, Epidemiology, and End Results.

^a^ Rural, nonmetropolitan counties (Rural/Urban Continuum codes 4–9); urban, metropolitan counties (Rural/Urban Continuum codes 1–3).

^b^ First quartile, 0%–0.40%; second quartile, 0.41%–1.98%; third quartile, 1.99%–10.53%; and fourth quartile, 10.54%–86.65%.

^c^ Non‐Hispanic American Indian/Alaska Native and unknown.

Elevated risks of CVD mortality were observed among cancer survivors residing in rural counties, counties in Q4 of Black residents, and counties with persistent poverty (Table 2). Compared with those in urban counties (SMR = 1.09; 95% CI = 1.08, 1.09), residents in rural counties had a significantly higher risk of CVD death (SMR = 1.27; 95% CI = 1.26, 1.28). Similarly, cancer survivors residing in counties with the highest quartile of Black residents had the highest SMR for CVD mortality (SMR = 1.15; 95% CI = 1.15, 1.16), while those in Q2 counties had the lowest SMR (SMR = 1.06; 95% CI = 1.05, 1.07). Furthermore, individuals in persistent poverty counties experienced a significantly elevated risk of CVD death (SMR = 1.35; 95% CI = 1.34, 1.37) compared with those in counties without persistent poverty (SMR = 1.09; 95% CI = 1.09, 1.10).

**TABLE 2:** Cardiovascular mortality risk among people with cancer diagnosis by county attribute, SEER 17, 2000–2021.

County attributes	Observed deaths, no.	Expected deaths, no.	SMR, observed vs. expected (95% CI)	EAR (per 10,000)	Persons with cancer, no.	Person‐years at risk
Rural/urban status^a^						
Rural	54,542	43,055	1.27 (1.26–1.28)	27.6	798,381	4,162,042
Urban	339,913	312,335	1.09 (1.08–1.09)	8.5	5,667,395	32,276,200
Persistent poverty status						
Persistent poverty	28,076	20,764	1.35 (1.34–1.37)	35.1	415,048	2,082,499
Non‐persistent poverty	366,379	334,627	1.09 (1.09–1.10)	9.2	6,050,728	34,355,743
% of Black residents quartile^b^						
First (lowest)	9931	9138	1.09 (1.07–1.11)	9.8	148,216	812,471
Second	44,279	41,597	1.06 (1.05–1.07)	6.3	760,545	4,279,038
Third	176,096	162,470	1.08 (1.08–1.09)	8.1	2,939,155	16,790,078
Fourth (highest)	164,149	142,186	1.15 (1.15–1.16)	15.1	2,617,866	14,556,663

Abbreviations: CI, confidence interval; EAR, excess absolute risk; No., number; SEER, Surveillance, Epidemiology, and End Results.

^a^ Rural, nonmetropolitan counties (Rural/Urban Continuum codes 4–9); urban, metropolitan counties (Rural/Urban Continuum codes 1–3).

^b^ First quartile, 0%–0.40%; second quartile, 0.41%–1.98%; third quartile, 1.99%–10.53%; and fourth quartile, 10.54%–86.65%.

**FIGURE 1:**
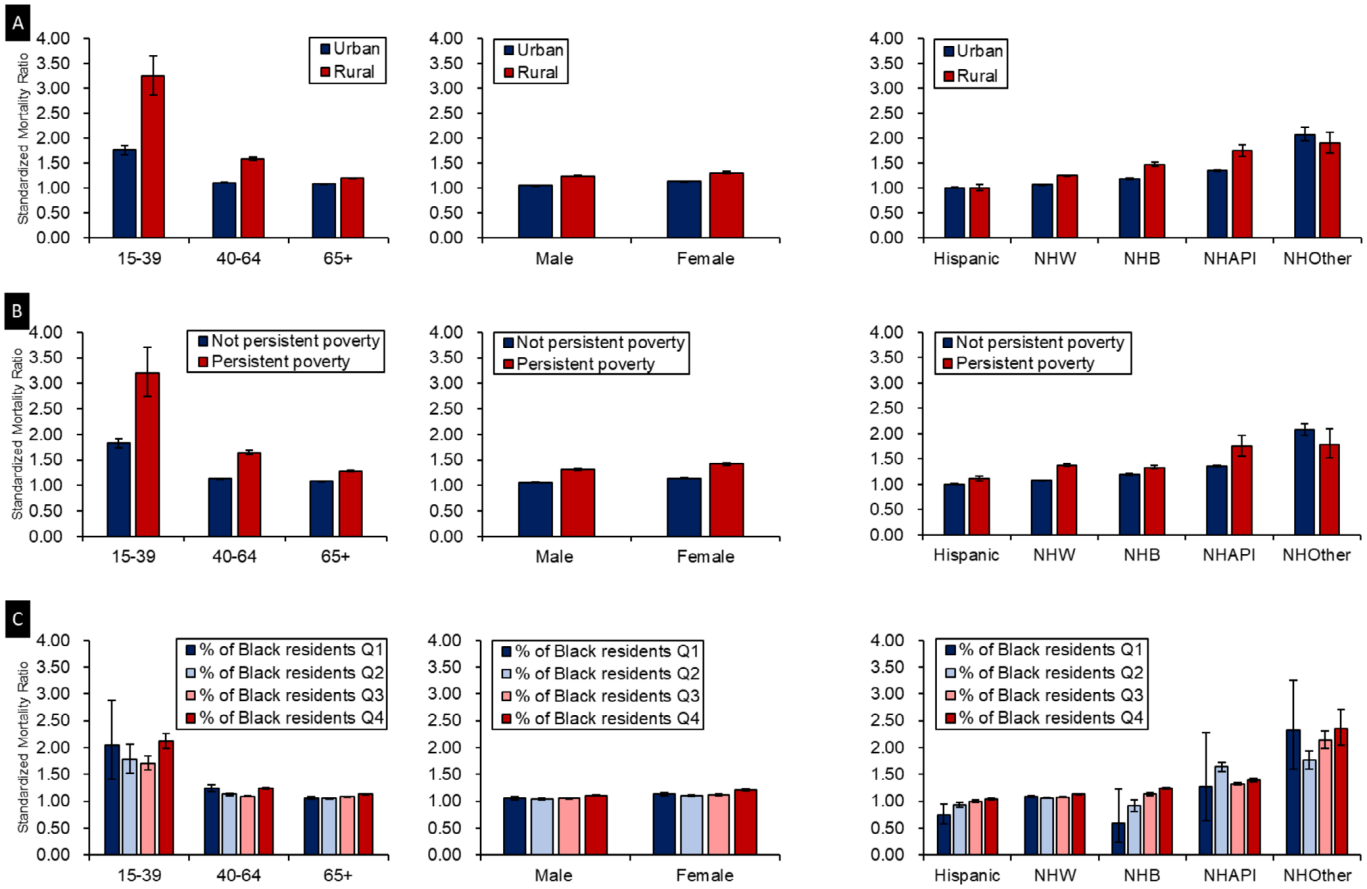
Standardized mortality ratios (SMRs) of cardiovascular disease (CVD) according to county‐level attributes by age, sex, and race and ethnicity: Surveillance, Epidemiology, and End Results 17 (SEER 17: 2000–2021). Panel A: CVD‐specific SMRs by rural/urban status across individual characteristics. Panel B: CVD‐specific SMRs by persistent poverty status across individual characteristics. Panel C: CVD‐specific SMRs by Black resident composition across individual characteristics.

When we further stratified by individual‐level, the observed patterns across county‐level characteristics were largely consistent across age groups, sex, and race and ethnicity (Figure 1). However, some variations were noted. Disparities in CVD mortality between county‐level attributes were most pronounced among individuals aged 15–39 years, compared with individuals diagnosed at older age, although the overall patterns were consistent across age groups. Women, compared with men, showed greater differences in CVD mortality across county‐level urbanization, racial composition, and persistent poverty status, although the pattern of risk was similar.

Among major non‐Hispanic racial groups, SMRs for CVD were significantly higher in rural counties and in those with persistent poverty compared with their respective counterparts (Figure 1A,B). However, the magnitude of EAR varied by racial group (Table S2). The difference between rural and urban counties was greatest among non‐Hispanic Black individuals (33.4 per 10,000), followed by non‐Hispanic API (28.8 per 10,000) and non‐Hispanic White (19.1 per 10,000) individuals. In contrast, the difference between counties with and without persistent poverty was largest among non‐Hispanic White individuals (30.8 per 10,000), with smaller gaps observed among non‐Hispanic API (23.3 per 10,000) and non‐Hispanic Black (16.7 per 10,000) individuals. Notably, Hispanic cancer survivors living in counties with persistent poverty had a significantly elevated risk of CVD mortality compared with the general population. In comparison, those living in counties without persistent poverty had CVD mortality risks similar to those of the general population. When cross‐stratified by county‐level racial composition and individual race and ethnicity, Hispanic individuals residing in counties with the lowest two quartiles of Black population proportion (Q1 and Q2) had significantly lower CVD mortality risks than the general population. Furthermore, SMRs increased progressively with the percentage of Black residents in the county among Hispanic, non‐Hispanic White, and non‐Hispanic Black groups. The most pronounced increase was observed among non‐Hispanic Black individuals, who exhibited the steepest gradient in CVD mortality risk with higher county‐level percentages of Black residents (Figure 1C).

**FIGURE 2:**
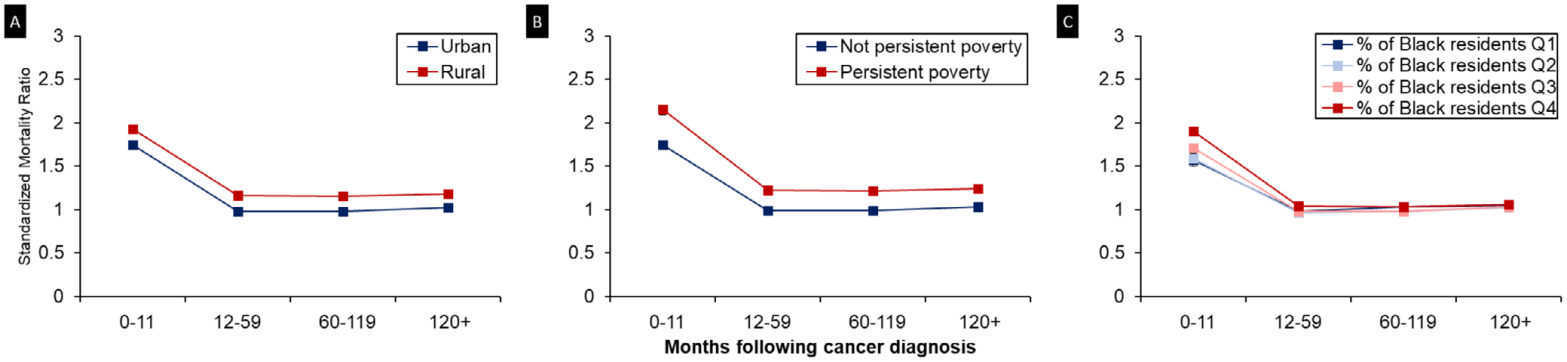
Cardiovascular mortality risk by latency period and county attribute: Surveillance, Epidemiology, and End Results 17 (SEER 17: 2000–2021). Panel A: CVD‐specific SMRs by rural/urban status over latency period following cancer diagnosis. Panel B: CVD‐specific SMRs by persistent poverty status over latency period following cancer diagnosis. Panel C: CVD‐specific SMRs by Black resident composition over latency period following cancer diagnosis. Error bars represent 95% confidence intervals (CIs). CIs are narrow and may not be visually distinguishable at this scale; exact values are provided in Table S3.

Regardless of county‐level attributes, the risk of CVD mortality was highest within the first year following a cancer diagnosis (Figure 2). Across all county groups, CVD risk declined after the first year but increased again 10 years post diagnosis. Notably, starting from year 2 post diagnosis, cancer survivors in counties without persistent poverty did not exhibit an elevated risk of CVD mortality within the first 10 years. In contrast, those residing in persistent poverty counties experienced a persistently elevated risk of CVD death throughout the follow‐up period, with an overall SMR of 1.35 (95% CI = 1.34, 1.37). A similar temporal pattern was observed when stratified by urban–rural status.

CVD risk among people diagnosed with cancer was higher in counties with lower educational attainment, lower median household income, and higher unemployment rate (Tables S4–S6). Also, when we cross‐stratified by rurality and persistent poverty status, cancer survivors living in rural and persistent poverty counties had the highest CVD mortality risk compared to all other groups (SMR = 1.53; 95% CI = 1.51, 1.56); in addition, the discrepancy in CVD risk among cancer survivors living in persistent poverty counties versus non‐persistent poverty counties was significantly higher in rural counties compared with urban counties (Table S7).

Cancer type analyses revealed substantial heterogeneity in county‐level disparities in CVD mortality. For rural–urban comparisons, the largest differences in EARs were observed among survivors of lung and bronchus cancer (42.9 per 10,000), followed by corpus uteri cancer (20.9 per 10,000) and prostate cancer (19.7 per 10,000). For comparisons by county‐level poverty, disparities were greatest among survivors of lung and bronchus cancer (51.3 per 10,000), followed by urinary bladder cancer (31.2 per 10,000) and prostate cancer (25.7 per 10,000). Across all examined cancer types, EARs and SMRs were consistently higher in rural and persistently impoverished counties compared with their respective reference groups (Table S8).

## 4 Discussion

In this study, we observed elevated CVD mortality risk following cancer diagnosis, predominantly among survivors living in rural counties, and counties with persistent poverty and very high percentage of Black residents. These patterns are similar to CVD trends in the general population [32, 33, 34]. Remarkably, non‐Hispanic API cancer survivors exhibited a uniformly increased CVD risk across all examined county attributes, a similar pattern was also observed in the general population [35]. This excess risk might be attributed to the heterogeneity within the people of API descent. For instance, a retrospective cohort study noted that Hawaiian breast cancer patients exhibited a higher hazard of CVD mortality compared with non‐Hispanic White counterparts, despite the overall lower CVD‐related mortality rates among the broader API patients [36]. In addition, factors such as acculturation and duration of residence in the US have been linked to adverse CVD risk factors and outcomes [37]. However, these within‐group differences in cardiovascular risk are often masked by clinical studies and surveys that aggregate API individuals into a single group. In contrast, the risk of CVD death following cancer diagnosis among Hispanic individuals did not significantly differ from that of the general population. This observation can be partially explained by the Hispanic health paradox, a well‐documented epidemiological phenomenon [38]. Notably, in this study, county‐level disparities in CVD mortality were most prominent among non‐Hispanic API individuals, women, and those aged 15–39 years, particularly when stratified by rural–urban status and persistent poverty.

The assessment of county‐level socioeconomic characteristics as a social determinant of health reflects conditions that affect all residents within the same county. Racial composition, as an ecological factor, is historically linked to area‐level affluence [39, 40]. Our study noted that the percentage of Black residents at the county level was significantly correlated with CVD‐specific mortality, particularly among non‐Hispanic Black and non‐Hispanic API cancer survivors. These findings suggest that racial residential segregation might be intricately associated with reduced access to healthcare and a lower quality of life among racially minoritized groups. Individuals living in rural areas exhibited an increased risk of CVD mortality following a cancer diagnosis compared with their urban counterparts, potentially due to a shortage of healthcare facilities and providers [41].

The role of persistent poverty in health outcomes among cancer survivors has only recently been recognized in the literature, and our findings align with this emerging body of research. Counties with persistent poverty often have a higher concentration of the racially minoritized population [42] and are predominantly rural [43]. Moss et al. [44] reported that patients living in counties with persistent poverty experienced 8.3 more deaths per 100,000 person‐years from cancer compared with those in counties without persistent poverty, after controlling for other county‐level characteristics. Furthermore, a cohort study that included patients with breast, colorectal, and lung cancers found that those in areas of persistent poverty were more likely to present at an advanced stage of cancer, less likely to receive surgical treatment, and had higher cancer‐specific mortality compared with their counterparts [45]. Similarly, Chen et al. [46] noted increased all‐cause and cancer‐specific mortality among breast cancer patients living in persistent poverty. Our research adds to the existing literature, demonstrating that individuals residing in persistent poverty counties face heightened CVD mortality risk following cancer diagnosis even more than a decade later compared with those in counties without persistent poverty. The disparities were even more pronounced in rural counties, where access to healthcare and insurance coverage are typically lower [22]. The mechanisms by which living in persistent poverty areas contributes to increased CVD mortality risk warrant further investigation.

In addition to geographic disparities, CVD mortality varied across age groups and sexes, indicating that factors beyond healthcare access contribute to these patterns. While limited access to specialty and preventive care in rural and persistently impoverished counties remains important, the age‐ and sex‐specific gradients observed here suggest a multifactorial etiology. Younger cancer survivors typically have lower baseline cardiovascular risk [47]. Thus, treatment‐related cardiotoxicity may represent a larger proportional increase in risk, resulting in higher relative mortality estimates. Younger patients may also be more likely to receive aggressive or dose‐intensive therapies with established cardiotoxic effects [48, 49, 50]. Sex‐specific differences in myocardial vulnerability, hormonal milieu, and pharmacokinetics may further increase susceptibility to cardiovascular injury among women [51, 52]. Comorbidity burden and health behaviors may also modify risk. Rural and persistently impoverished areas have higher prevalence of smoking, obesity, physical inactivity, hypertension, and diabetes, which may interact with treatment‐related cardiac injury [53, 54]. Finally, disparities in cardiovascular screening and symptom recognition may amplify these patterns, as CVD is more likely to be under‐recognized in women and less routinely monitored in younger survivors, particularly in under‐resourced settings [55].

We also noted that CVD mortality risk peaked in the first year after cancer diagnosis across all county‐level strata. This aligns with large population‐based analyses of cancer survivors [56], and supports clinical guidelines on cardio‐oncology that designate the treatment phase and the subsequent 12 months as the period requiring the most intensive cardiovascular surveillance [57]. The CVD risk then dropped significantly and remained stable during the 1–5 years post diagnosis. Beyond 5 years since diagnosis, the risk moderately increased, with rural and persistent poverty counties exhibiting consistently higher risks compared with their counterparts. Aging, an established independent risk factor for CVD [58, 59, 60], may contribute to this pattern, further compounded by the cumulative effects of the physical and built environments in socially deprived areas. For instance, rural areas, despite having more green space, often feature significantly lower walkability than urban settings [61]. Additional barriers in the built environment, such as lack of community support and low level of perceived safety, are negatively associated with healthy aging [62, 63, 64]. A recent study found that only 4% of cancer survivors adhered to all four American Cancer Society guidelines for nutrition and physical activity, highlighting the widespread challenges in adopting and maintaining heart‐healthy behaviors in survivorship [65]. These challenges may be further amplified in under‐resourced communities, contributing to elevated CVD risk among cancer survivors, particularly in rural or persistently impoverished areas.

The magnitude of county‐level disparities varied by cancer type, with particularly pronounced differences for lung and bronchus cancer, followed by prostate, urinary bladder, and corpus uteri cancers. These patterns likely reflect differences in baseline cardiovascular risk, comorbidity burden, survivorship care access, and continuity of cardiovascular risk management across cancer populations [66]. The prominence of lung and bronchus cancer survivors is consistent with their high prevalence of shared risk factors for both cancer and CVD, while the observed variations among survivors of other cancers suggest that geographic and socioeconomic variations in cardiovascular mortality are substantial across cancer sites.

This study has limitations. First, cause of death information derived from death certificates is subject to misclassification, particularly among older adults and individuals with multiple comorbidities [67]. In cancer survivors, distinguishing cardiovascular causes of death from cancer‐ or treatment‐related mortality can be challenging, potentially leading to under‐ or over‐estimation of CVD‐specific mortality. However, such misclassification is unlikely to differ systematically by county‐level characteristics and would be expected to bias estimates toward the null. Second, the SEER database restricts to county‐level characteristics, which may not capture the nuances within large and heterogeneous counties as effectively as they might in smaller, more homogeneous counties. Third, using the county‐level percentage of Black residents as a proxy for residential segregation is a crude measure, and its validity is questionable because a high percentage of a single racial group does not necessarily equate to high segregation [68]. Furthermore, our analysis was limited to a few county characteristics supported by the National Cancer Institute. Future research should expand to include other area‐level socioeconomic attributes to better understand their roles in CVD risk in people diagnosed with cancer. Despite these limitations, our study contributes to the existing literature by examining CVD mortality after cancer diagnosis in relation to various area‐level social determinants of health. Given the nationally representative nature of the cancer registry data, our findings are generalizable to the general US population.

## 5 Conclusions

In the US, while overall CVD risks are higher among cancer survivors compared with the general population, those residing in rural and persistently impoverished counties experience an even greater risk. Our results underscore the need to improve cardiovascular care among cancer survivors, particularly in socially vulnerable communities, including expanding access to specialized cardio‐oncology services and promoting integrative care that emphasizes nutrition and physical activity.

## Author Contributions

**Yenan Zhu:** conceptualization (equal), formal analysis (lead), methodology (equal), visualization (equal), writing – original draft (lead). **Ryan Suk:** conceptualization (equal), methodology (equal), writing – review and editing (equal). **Yueh‐Yun Lin:** visualization (equal), writing – review and editing (equal). **Young‐Rock Hong:** writing – review and editing (equal). **Beth Virnig:** funding acquisition (lead), supervision (lead), writing – review and editing (equal).

## Funding

The authors have nothing to report.

## Conflicts of Interest

The authors declare no conflicts of interest.

## Supporting information


**Table S1:** Codes used to identify cardiovascular disease–specific causes of death.
**Table S2:** Cardiovascular risk among people with cancer diagnosis, stratified by county‐level characteristics and by individual sociodemographic factors, SEER 17, 2000–2021.
**Table S3:** Standardized mortality ratios (SMRs) by latency period, stratified by county‐level characteristics, SEER 17, 2000–2021.
**Table S4:** Cardiovascular risk among people with cancer diagnosis, stratified by county‐level educational attainment, SEER 17, 2000–2021.
**Table S5:** Cardiovascular risk among people with cancer diagnosis, stratified by county‐level median household income, SEER 17, 2000–2021.
**Table S6:** Cardiovascular risk among people with cancer diagnosis, stratified by county‐level unemployment, SEER 17, 2000–2021.
**Table S7:** Cardiovascular risk among people with cancer diagnosis, cross‐stratified by county‐level rurality and persistent poverty status, SEER 17, 2000–2021.
**Table S8:** County‐level absolute risk differences in cardiovascular mortality by cancer type, SEER 17, 2000–2021.

## Data Availability

The data used in this study are publicly available from the National Cancer Institute's Surveillance, Epidemiology, and End Results (SEER) Program under a data use agreement.
