# Supplementary material for: Cardiovascular Mortality Risk After Cancer Diagnosis by County‐Level Characteristics in the United States 2000–2021
Source: Cancer Med. 2026 Feb 25;15(3):e71675. doi: 10.1002/cam4.71675 (PMC12935517; doi:10.1002/cam4.71675)
Supplement: Supplementary file 1 — Table S1: Codes used to identify cardiovascular disease–specific causes of death. Table S2: Cardiovascular risk among people with cancer diagnosis, stratified by county‐level characteristics and by individual sociodemographic factors, SEER 17, 2000–2021. Table S3: Standardized mortality ratios (SMRs) by latency period, stratified by county‐level characteristics, SEER 17, 2000–2021. Table S4: Cardiovascular risk among people with cancer diagnosis, stratified by county‐level educational attainment, SEER 17, 2000–2021. Table S5: Cardiovascular risk among people with cancer diagnosis, stratified by county‐level median household income, SEER 17, 2000–2021. Table S6: Cardiovascular risk among people with cancer diagnosis, stratified by county‐level unemployment, SEER 17, 2000–2021. Table S7: Cardiovascular risk among people with cancer diagnosis, cross‐stratified by county‐level rurality and persistent poverty status, SEER 17, 2000–2021. Table S8: County‐level absolute risk differences in cardiovascular mortality by cancer type, SEER 17, 2000–2021. [file CAM4-15-e71675-s001.docx]

**Supplement Table 1.** Codes Used to Identify Cardiovascular Disease–Specific Causes of Death

| **Cause of Death** | **ICD-9 code** | **ICD-10 code** | **Recode value** |
| --- | --- | --- | --- |
| Hypertensive disease | 400-404 | I10-I15 | 106 |
| Disease of heart | 410-417 | I20-I28 | 107, 108 |
| Cerebrovascular diseases | 430-438 | I60-I69 | 109 |
| Diseases of arteries, arterioles, capillaries | 440-448 | I70-I78 | 110 |

**Supplement Table 2.** Cardiovascular Risk Among People with Cancer Diagnosis, Stratified by County-Level Characteristics and by Individual Sociodemographic Factors, SEER 17, 2000-2021

| **County attributes** | **Observed deaths, No.** | **Expected deaths, No.** | **SMR, observed vs. expected (95%)** | **EAR (per 10,000)** | **Persons with cancer, No.** | **Person-years at risk** |
| --- | --- | --- | --- | --- | --- | --- |
| **Rural** |  |  |  |  |  |  |
| Age group |  |  |  |  |  |  |
| 15-39 years | 283 | 87 | 3.25 (2.88-3.65) | 6.8 | 35,843 | 287,282 |
| 40-64 years | 10,616 | 6,680 | 1.59 (1.56-1.62) | 19.1 | 328,348 | 2,065,985 |
| 65+ years | 43,643 | 36,288 | 1.20 (1.19-1.21) | 40.7 | 434,190 | 1,808,776 |
| Sex |  |  |  |  |  |  |
| Male | 32,453 | 26,173 | 1.24 (1.23-1.25) | 29.3 | 427,170 | 2,143,144 |
| Female | 22,089 | 16,882 | 1.31 (1.29-1.33) | 25.8 | 371,211 | 2,018,898 |
| Race and ethnicity |  |  |  |  |  |  |
| Hispanic | 1,198 | 1,186 | 1.01 (0.95-1.07) | 0.9 | 28,629 | 141,934 |
| Non-Hispanic |  |  |  |  |  |  |
| Asian/Pacific Islander | 809 | 461 | 1.76 (1.64-1.88) | 48.8 | 13,854 | 71,430 |
| Black | 4,685 | 3,173 | 1.48 (1.43-1.52) | 52.1 | 62,516 | 289,916 |
| White | 47,529 | 38,066 | 1.25 (1.24-1.26) | 26.1 | 685,346 | 3,620,329 |
| Other^c^ | 321 | 169 | 1.90 (1.70-2.12) | 39.5 | 8,036 | 38,432 |
| **Urban** |  |  |  |  |  |  |
| Age group |  |  |  |  |  |  |
| 15-39 years | 1,520 | 859 | 1.77 (1.68-1.86) | 2.4 | 350,786 | 2,773,655 |
| 40-64 years | 59,475 | 53,465 | 1.11 (1.10-1.12) | 3.6 | 2,467,314 | 16,845,317 |
| 65+ years | 278,918 | 258,012 | 1.08 (1.08-1.09) | 16.5 | 2,849,295 | 12,657,228 |
| Sex |  |  |  |  |  |  |
| Male | 197,826 | 187,445 | 1.06 (1.05-1.06) | 6.5 | 2,875,711 | 16,044,300 |
| Female | 142,087 | 124,891 | 1.14 (1.13-1.14) | 10.6 | 2,791,684 | 16,231,900 |
| Race and ethnicity |  |  |  |  |  |  |
| Hispanic | 26,489 | 26,231 | 1.01 (1.00-1.02) | 0.7 | 681,620 | 3,550,763 |
| Non-Hispanic |  |  |  |  |  |  |
| Asian/Pacific Islander | 18,385 | 13,582 | 1.35 (1.33-1.37) | 20.0 | 448,096 | 2,396,947 |
| Black | 36,730 | 30,963 | 1.19 (1.17-1.20) | 18.7 | 599,749 | 3,080,673 |
| White | 257,324 | 241,088 | 1.07 (1.06-1.07) | 7.0 | 3,915,598 | 23,133,734 |
| Other^c^ | 985 | 473 | 2.08 (1.96-2.22) | 44.9 | 22,332 | 114,084 |
| **Persistent poverty** |  |  |  |  |  |  |
| Age group |  |  |  |  |  |  |
| 15-39 years | 176 | 55 | 3.20 (2.75-3.71) | 7.3 | 22,223 | 165,854 |
| 40-64 years | 6,103 | 3,721 | 1.64 (1.60-1.68) | 22.3 | 180,149 | 1,066,629 |
| 65+ years | 21,797 | 16,988 | 1.28(1.27-1.30) | 56.6 | 212,676 | 850,016 |
| Sex |  |  |  |  |  |  |
| Male | 16,569 | 12,663 | 1.30 (1.29-1.33) | 37.0 | 218,824 | 1,054,844 |
| Female | 11,507 | 8,101 | 1.42 (1.39-1.45) | 33.1 | 196,224 | 1,027,655 |
| Race and ethnicity |  |  |  |  |  |  |
| Hispanic | 2,105 | 1,902 | 1.11 (1.06-1.16) | 9.0 | 45,904 | 225,989 |
| Non-Hispanic |  |  |  |  |  |  |
| Asian/Pacific Islander | 285 | 163 | 1.75 (1.55-1.96) | 43.9 | 6,172 | 27,758 |
| Black | 5,903 | 4,425 | 1.33 (1.30-1.37) | 36.3 | 86,305 | 407,241 |
| White | 19,622 | 14,184 | 1.38 (1.36-1.40) | 38.8 | 272,463 | 1,401,891 |
| Other^c^ | 161 | 90 | 1.79 (1.52-2.09) | 36.2 | 4,204 | 19,620 |
| **Non-persistent poverty** |  |  |  |  |  |  |
| Age group |  |  |  |  |  |  |
| 15-39 years | 1,627 | 891 | 1.83 (1.74-1.92) | 2.5 | 364,406 | 2,895,082 |
| 40-64 years | 63,988 | 56,424 | 1.13 (1.13-1.14) | 4.2 | 2,615,513 | 17,844,673 |
| 65+ years | 300,764 | 277,312 | 1.08 (1.08-1.09) | 17.2 | 3,070,809 | 13,615,988 |
| Sex |  |  |  |  |  |  |
| Male | 213,710 | 200,955 | 1.06 (1.06-1.07) | 7.4 | 3,084,057 | 17,132,600 |
| Female | 152,669 | 133,672 | 1.14 (1.14-1.15) | 11.0 | 2,966,671 | 17,223,144 |
| Race and ethnicity |  |  |  |  |  |  |
| Hispanic | 25,582 | 25,515 | 1.00 (0.99-1.01) | 0.2 | 664,345 | 3,466,709 |
| Non-Hispanic |  |  |  |  |  |  |
| Asian/Pacific Islander | 18,909 | 13,879 | 1.36 (1.34-1.38) | 20.6 | 455,778 | 2,440,619 |
| Black | 35,512 | 29,711 | 1.20 (1.18-1.21) | 19.6 | 575,960 | 2,963,348 |
| White | 285,231 | 264,970 | 1.08 (1.07-1.08) | 8.0 | 4,328,481 | 25,352,173 |
| Other^c^ | 1,145 | 552 | 2.08 (1.96-2.20) | 44.6 | 26,164 | 132,896 |
| **1^st^ quartile of Black residents** |  |  |  |  |  |  |
| Age group |  |  |  |  |  |  |
| 15-39 years | 33 | 16 | 2.05 (1.41-2.89) | 2.9 | 7,078 | 58,167 |
| 40-64 years | 1,469 | 1,184 | 1.24 (1.18-1.31) | 7.5 | 57,143 | 381,108 |
| 65+ years | 8,429 | 7,937 | 1.06 (1.04-1.08) | 13.2 | 83,995 | 373,196 |
| Sex |  |  |  |  |  |  |
| Male | 5,907 | 5,593 | 1.06 (1.03-1.08) | 7.4 | 80,120 | 427,398 |
| Female | 4,024 | 3,545 | 1.14 (1.10-1.17) | 12.5 | 68,096 | 385,073 |
| Race and ethnicity |  |  |  |  |  |  |
| Hispanic | 65 | 87 | 0.74 (0.57-0.95) | -19.9 | 2,286 | 11,222 |
| Non-Hispanic | 11 | 9 | 1.27 (0.64-2.28) | 11.8 | 427 | 1,990 |
| Asian/Pacific Islander | 7 | 12 | 0.59 (0.24-1.22) | -40.2 | 261 | 1,210 |
| Black | 9,815 | 9,015 | 1.09 (1.07-1.11) | 10.1 | 144,469 | 794,343 |
| White | 33 | 14 | 2.32 (1.60-3.26) | 50.7 | 773 | 3,706 |
| Other^c^ | 65 | 87 | 0.74 (0.57-0.95) | -19.9 | 2,286 | 11,222 |
| **2^nd^ quartile of Black residents** |  |  |  |  |  |  |
| Age group |  |  |  |  |  |  |
| 15-39 years | 171 | 96 | 1.78 (1.52-2.06) | 2.1 | 43,390 | 352,589 |
| 40-64 years | 7,415 | 6,572 | 1.13 (1.10-1.15) | 3.9 | 318,384 | 2,173,144 |
| 65+ years | 36,693 | 34,928 | 1.05 (1.04-1.06) | 10.1 | 398,771 | 1,753,305 |
| Sex |  |  |  |  |  |  |
| Male | 26,476 | 25,532 | 1.04 (1.02-1.05) | 4.3 | 397,558 | 2,175,555 |
| Female | 17,803 | 16,065 | 1.11 (1.09-1.12) | 8.3 | 362,987 | 2,103,483 |
| Race and ethnicity |  |  |  |  |  |  |
| Hispanic | 1,895 | 2,027 | 0.94 (0.89-0.98) | -5.0 | 50,733 | 264,042 |
| Non-Hispanic | 1,380 | 844 | 1.64 (1.55-1.72) | 37.8 | 27,552 | 141,853 |
| Asian/Pacific Islander | 282 | 309 | 0.91 (0.81-1.03) | -8.6 | 6,234 | 31,656 |
| Black | 40,304 | 38,181 | 1.06 (1.05-1.07) | 5.6 | 665,011 | 3,786,675 |
| White | 418 | 237 | 1.77 (1.60-1.94) | 33.1 | 11,015 | 54,811 |
| Other^c^ | 171 | 96 | 1.78 (1.52-2.06) | 2.1 | 43,390 | 352,589 |
| **3^rd^ quartile of Black residents** |  |  |  |  |  |  |
| Age group |  |  |  |  |  |  |
| 15-39 years | 704 | 413 | 1.71 (1.58-1.84) | 2.1 | 178,305 | 1,419,935 |
| 40-64 years | 28,543 | 26,122 | 1.09 (1.08-1.11) | 2.8 | 1,258,074 | 8,676,533 |
| 65+ years | 146,849 | 135,935 | 1.08 (1.07-1.09) | 16.3 | 1,502,776 | 6,693,610 |
| Sex |  |  |  |  |  |  |
| Male | 102,732 | 97,141 | 1.06 (1.05-1.06) | 6.7 | 1,494,448 | 8,322,381 |
| Female | 73,364 | 65,329 | 1.12 (1.11-1.13) | 9.5 | 1,444,707 | 8,467,697 |
| Race and ethnicity |  |  |  |  |  |  |
| Hispanic | 13,803 | 13,793 | 1.00 (0.98-1.02) | 0.1 | 363,815 | 1,873,456 |
| Non-Hispanic | 11,121 | 8,418 | 1.32 (1.30-1.35) | 18.5 | 271,368 | 1,461,590 |
| Asian/Pacific Islander | 7,960 | 7,042 | 1.13 (1.11-1.16) | 13.1 | 133,191 | 702,539 |
| Black | 142,558 | 132,912 | 1.07 (1.07-1.08) | 7.6 | 2,156,495 | 12,679,630 |
| White | 654 | 306 | 2.14 (1.98-2.31) | 47.8 | 14,286 | 72,863 |
| Other^c^ | 704 | 413 | 1.71 (1.58-1.84) | 2.1 | 178,305 | 1,419,935 |
| **4^th^ quartile of Black residents** |  |  |  |  |  |  |
| Age group |  |  |  |  |  |  |
| 15-39 years | 895 | 421 | 2.13 (1.99-2.27) | 3.9 | 157,857 | 1,230,247 |
| 40-64 years | 32,664 | 26,266 | 1.24 (1.23-1.26) | 8.3 | 1,162,063 | 7,680,521 |
| 65+ years | 130,590 | 115,499 | 1.13 (1.12-1.14) | 26.7 | 1,297,946 | 5,645,895 |
| Sex |  |  |  |  |  |  |
| Male | 95,164 | 85,352 | 1.11 (1.11-1.12) | 13.5 | 1,330,758 | 7,262,113 |
| Female | 68,985 | 56,834 | 1.21 (1.20-1.22) | 16.7 | 1,287,108 | 7,294,550 |
| Race and ethnicity |  |  |  |  |  |  |
| Hispanic | 11,924 | 11,510 | 1.04 (1.02-1.05) | 2.7 | 293,415 | 1,543,977 |
| Non-Hispanic | 6,682 | 4,772 | 1.40 (1.37-1.43) | 22.1 | 162,605 | 862,947 |
| Asian/Pacific Islander | 33,166 | 26,773 | 1.24 (1.23-1.25) | 24.3 | 522,579 | 2,635,184 |
| Black | 112,176 | 99,046 | 1.13 (1.13-1.14) | 13.8 | 1,634,973 | 9,493,419 |
| White | 201 | 85 | 2.36 (2.04-2.71) | 54.8 | 4,294 | 21,136 |
| Other^c^ | 895 | 421 | 2.13 (1.99-2.27) | 3.9 | 157,857 | 1,230,247 |

Abbreviations: CI, confidence interval; EAR, excess absolute risk; No., number; SEER, Surveillance, Epidemiology, and End Results; SMR, standardized mortality ratio.

First quartile, 0%-0.40%; second quartile, 0.41%-1.98%; third quartile, 1.99%-10.53%; and fourth quartile, 10.54%-86.65%.

Non-Hispanic American Indian/Alaska Native and unknown.

**Supplement Table 3.** Standardized Mortality Ratios (SMRs) by Latency Period, Stratified by County-Level Characteristics, SEER 17, 2000-2021

| **Latency** | **Observed deaths, No.** | **Expected deaths, No.** | **SMR, observed vs. expected (95%)** | **EAR (per 10,000)** | **Persons with cancer, No.** | **Person-years at risk** |
| --- | --- | --- | --- | --- | --- | --- |
| **Overall** |  |  |  |  |  |  |
| 0-11 months | 81,957 | 46,381 | 1.77 (1.75-1.78) | 66.1 | 6,467,098 | 5,382,041 |
| 12-59 months | 125,681 | 125,484 | 1.00 (1.00-1.01) | 0.1 | 4,796,196 | 14,382,191 |
| 60-119 months | 103,517 | 103,307 | 1.00 (1.00-1.01) | 0.2 | 2,762,270 | 10,268,302 |
| 120+ months | 83,385 | 80,318 | 1.04 (1.03-1.05) | 4.8 | 1,448,831 | 6,414,300 |
| **Rural** |  |  |  |  |  |  |
| 0-11 months | 11,387 | 5,910 | 1.93 (1.89-1.96) | 84.6 | 798,381 | 647,399 |
| 12-59 months | 18,030 | 15,573 | 1.16 (1.14-1.17) | 14.7 | 568,200 | 1,669,578 |
| 60-119 months | 14,294 | 12,403 | 1.15 (1.13-1.17) | 16.4 | 315,604 | 1,154,895 |
| 120+ months | 10,831 | 9,170 | 1.18 (1.16-1.20) | 24.1 | 160,146 | 690,169 |
| **Urban** |  |  |  |  |  |  |
| 0-11 months | 70,557 | 40,460 | 1.74 (1.73-1.76) | 63.6 | 5,667,395 | 4,733,585 |
| 12-59 months | 107,626 | 109,878 | 0.98 (0.97-0.99) | -1.8 | 4,227,004 | 12,709,400 |
| 60-119 months | 89,193 | 90,872 | 0.98 (0.98-0.99) | -1.8 | 2,445,990 | 9,110,664 |
| 120+ months | 72,537 | 71,126 | 1.02 (1.01-1.03) | 2.5 | 1,288,289 | 5,722,552 |
| **Persistent poverty** |  |  |  |  |  |  |
| 0-11 months | 6,261 | 2,917 | 2.15 (2.09-2.20) | 100.7 | 415,048 | 332,277 |
| 12-59 months | 9,261 | 7,568 | 1.22 (1.20-1.25) | 20.1 | 289,615 | 841,004 |
| 60-119 months | 7,212 | 5,957 | 1.21 (1.18-1.24) | 22.0 | 157,417 | 571,079 |
| 120+ months | 5,342 | 4,323 | 1.24 (1.20-1.27) | 30.2 | 78,281 | 338,140 |
| **Non-persistent poverty** |  |  |  |  |  |  |
| 0-11 months | 75,683 | 43,454 | 1.74 (1.73-1.75) | 63.8 | 6,050,728 | 5,048,707 |
| 12-59 months | 116,395 | 117,882 | 0.99 (0.98-0.99) | -1.1 | 4,505,589 | 13,537,974 |
| 60-119 months | 96,275 | 97,318 | 0.99 (0.98-1.00) | -1.1 | 2,604,177 | 9,694,481 |
| 120+ months | 78,026 | 75,973 | 1.03 (1.02-1.03) | 3.4 | 1,370,154 | 6,074,582 |
| **1^st^ quartile of Black residents** |  |  |  |  |  |  |
| 0-11 months | 1,903 | 1,213 | 1.57 (1.50-1.64) | 56.2 | 148,216 | 122,830 |
| 12-59 months | 3,218 | 3,288 | 0.98 (0.95-1.01) | -2.2 | 109,108 | 325,055 |
| 60-119 months | 2,737 | 2,654 | 1.03 (0.99-1.07) | 3.6 | 61,904 | 227,426 |
| 120+ months | 2,073 | 1,982 | 1.05 (1.00-1.09) | 6.6 | 31,726 | 137,160 |
| **2^nd^ quartile of Black residents** |  |  |  |  |  |  |
| 0-11 months | 8,629 | 5,420 | 1.59 (1.56-1.63) | 50.7 | 760,545 | 633,004 |
| 12-59 months | 14,194 | 14,733 | 0.96 (0.95-0.98) | -3.2 | 564,500 | 1,697,448 |
| 60-119 months | 11,923 | 12,134 | 0.98 (0.97-1.00) | -1.7 | 325,911 | 1,208,144 |
| 120+ months | 9,533 | 9,310 | 1.02 (1.00-1.04) | 3.0 | 169,753 | 740,442 |
| **3^rd^ quartile of Black residents** |  |  |  |  |  |  |
| 0-11 months | 35,905 | 21,017 | 1.71 (1.69-1.73) | 60.6 | 2,939,155 | 2,458,190 |
| 12-59 months | 55,974 | 57,151 | 0.98 (0.97-0.99) | -1.8 | 2,197,072 | 6,610,750 |
| 60-119 months | 46,243 | 47,286 | 0.98 (0.97-0.99) | -2.2 | 1,273,071 | 4,740,910 |
| 120+ months | 37,974 | 37,017 | 1.03 (1.02-1.04) | 3.2 | 670,523 | 2,980,228 |
| **4^th^ quartile of Black residents** |  |  |  |  |  |  |
| 0-11 months | 35,507 | 18,720 | 1.90 (1.88-1.92) | 77.5 | 2,617,866 | 2,166,964 |
| 12-59 months | 52,270 | 50,279 | 1.04 (1.03-1.05) | 3.5 | 1,924,528 | 5,745,728 |
| 60-119 months | 42,584 | 41,201 | 1.03 (1.02-1.04) | 3.4 | 1,100,708 | 4,089,080 |
| 120+ months | 33,788 | 31,987 | 1.06 (1.05-1.07) | 7.1 | 576,433 | 2,554,891 |

Abbreviations: CI, confidence interval; EAR, excess absolute risk; No., number; SEER, Surveillance, Epidemiology, and End Results; SMR, standardized mortality ratio.

First quartile, 0%-0.40%; second quartile, 0.41%-1.98%; third quartile, 1.99%-10.53%; and fourth quartile, 10.54%-86.65%.

Non-Hispanic American Indian/Alaska Native and unknown.

**Supplement Table 4.** Cardiovascular Risk Among People with Cancer Diagnosis, Stratified by County-Level Educational Attainment, SEER 17, 2000-2021

| **County attributes** | **Observed deaths, No.** | **Expected deaths, No.** | **SMR, observed vs. expected (95%)** | **EAR (per 10,000)** | **Persons with cancer, No.** | **Person-years at risk** |
| --- | --- | --- | --- | --- | --- | --- |
| **% of less than high school education** |  |  |  |  |  |  |
| First (lowest) quartile | 114,309 | 116,213 | 0.98 (0.98-0.99) | -1.6 | 2,040,245 | 12,156,671 |
| Second quartile | 124,319 | 116,699 | 1.07 (1.06-1.07) | 6.5 | 2,057,773 | 11,703,222 |
| Third quartile | 57,736 | 46,519 | 1.24 (1.23-1.25) | 23.8 | 889,853 | 4,711,257 |
| Fourth (Highest) quartile | 98,091 | 75,961 | 1.29 (1.28-1.30) | 28.1 | 1,477,911 | 7,867,100 |

Abbreviations: CI, confidence interval; EAR, excess absolute risk; No., number; SEER, Surveillance, Epidemiology, and End Results; SMR, standardized mortality ratio.

First quartile, 3.04%-15.91%; second quartile, 15.92%-20.76%; third quartile, 20.77%-28.66%; and fourth quartile, 28.67%-65.30%.

**Supplement Table 5.** Cardiovascular Risk Among People with Cancer Diagnosis, Stratified by County-Level Median Household Income, SEER 17, 2000-2021

| **County attributes** | **Observed deaths, No.** | **Expected deaths, No.** | **SMR, observed vs. expected (95%)** | **EAR (per 10,000)** | **Persons with cancer, No.** | **Person-years at risk** |
| --- | --- | --- | --- | --- | --- | --- |
| **Median household income** |  |  |  |  |  |  |
| First (lowest) quartile | 25,720 | 18,449 | 1.39 (1.38-1.41) | 38.9 | 379,092 | 1,869,438 |
| Second quartile | 25,000 | 19,907 | 1.26 (1.24-1.27) | 26.2 | 377,625 | 1,944,304 |
| Third quartile | 58,715 | 48,387 | 1.21 (1.20-1.22) | 21.6 | 891,344 | 4,776,842 |
| Fourth (Highest) quartile | 285,020 | 268,649 | 1.06 (1.06-1.06) | 5.9 | 4,817,721 | 27,847,667 |

Abbreviations: CI, confidence interval; EAR, excess absolute risk; No., number; SEER, Surveillance, Epidemiology, and End Results; SMR, standardized mortality ratio.

First quartile, $9,330-$29,699; second quartile, $29,700-$33,849; third quartile, $33,850-$39,539; and fourth quartile, $39,540-$82,939.

**Supplement Table 6.** Cardiovascular Risk Among People with Cancer Diagnosis, Stratified by County-Level Unemployment, SEER 17, 2000-2021

| **County attributes** | **Observed deaths, No.** | **Expected deaths, No.** | **SMR, observed vs. expected (95%)** | **EAR (per 10,000)** | **Persons with cancer, No.** | **Person-years at risk** |
| --- | --- | --- | --- | --- | --- | --- |
| **% unemployed** |  |  |  |  |  |  |
| First (lowest) quartile | 49,048 | 48,311 | 1.02 (1.01-1.02) | 1.4 | 893,828 | 5,222,945 |
| Second quartile | 101,880 | 98,385 | 1.04 (1.03-1.04) | 3.5 | 1,710,142 | 9,986,683 |
| Third quartile | 102,558 | 93,194 | 1.10 (1.09-1.11) | 9.8 | 1,703,312 | 9,567,912 |
| Fourth (Highest) quartile | 140,969 | 115,502 | 1.22 (1.21-1.23) | 21.8 | 2,158,500 | 11,660,711 |

Abbreviations: CI, confidence interval; EAR, excess absolute risk; No., number; SEER, Surveillance, Epidemiology, and End Results; SMR, standardized mortality ratio.

First quartile, 0%-3.97%; second quartile, 3.98%-5.35%; third quartile, 5.36%-6.94%; and fourth quartile, 6.95%-41.67%.

**Supplement Table 7.** Cardiovascular Risk Among People with Cancer Diagnosis, Cross-Stratified by County-Level Rurality and Persistent Poverty Status, SEER 17, 2000-2021

| **County attributes** | **Observed deaths, No.** | **Expected deaths, No.** | **SMR, observed vs. expected (95%)** | **EAR (per 10,000)** | **Persons with cancer, No.** | **Person-years at risk** |
| --- | --- | --- | --- | --- | --- | --- |
| **Rural** |  |  |  |  |  |  |
| Persistent poverty | 13,275 | 8,670 | 1.53 (1.51-1.56) | 50.3 | 190,536 | 914,827 |
| Non-persistent poverty | 41,267 | 34,386 | 1.20 (1.19-1.21) | 21.2 | 607,845 | 3,247,215 |
| **Urban** |  |  |  |  |  |  |
| Persistent poverty | 14,801 | 12,095 | 1.22 (1.20-1.24) | 23.2 | 224,512 | 1,167,672 |
| Non-persistent poverty | 325,112 | 300,241 | 1.08 (1.08-1.09) | 8.0 | 5,442,883 | 31,108,529 |

Abbreviations: CI, confidence interval; EAR, excess absolute risk; No., number; SEER, Surveillance, Epidemiology, and End Results; SMR, standardized mortality ratio.

**Supplement Table 8.** County-Level Absolute Risk Differences in Cardiovascular Mortality by Cancer Type, SEER 17, 2000-2021

| **Cancer type** | **Rural EAR**  **(per 10,000)** | **Urban EAR**  **(per 10,000)** | **Rural-urban**  **EAR difference** | **Persistent poverty EAR (per 10,000)** | **Non-persistent poverty EAR (per 10,000)** | **Poverty EAR difference** |
| --- | --- | --- | --- | --- | --- | --- |
| Breast | 3.98 | -7.44 | 11.42 | 8.33 | -7.09 | 15.42 |
| Prostate | -14.44 | -34.09 | 19.65 | -7.71 | -33.38 | 25.67 |
| Lung and bronchus | 139.92 | 97.03 | 42.89 | 150.92 | 99.64 | 51.28 |
| Colon and rectum | 35.43 | 21.75 | 13.68 | 40.40 | 22.33 | 18.07 |
| Urinary bladder | 39.48 | 23.30 | 16.18 | 54.95 | 23.74 | 31.21 |
| Corpus Uteri | 22.72 | 1.80 | 20.92 | 28.09 | 2.88 | 25.21 |
| Kidney and renal pelvis | 41.13 | 25.96 | 15.17 | 44.79 | 26.59 | 18.20 |

Abbreviations: CI, confidence interval; EAR, excess absolute risk; No., number; SEER, Surveillance, Epidemiology, and End Results.
